# Near‐infrared spectroscopy for metabolite quantification and species identification

**DOI:** 10.1002/ece3.4847

**Published:** 2019-01-13

**Authors:** Wen C. Aw, John William O. Ballard

**Affiliations:** ^1^ School of Biotechnology and Biomolecular Sciences University of New South Wales Sydney New South Wales Australia

**Keywords:** ecology, high‐throughput, metabolite level, noninvasive, species identification

## Abstract

Near‐infrared (NIR) spectroscopy is a high‐throughput method to analyze the near‐infrared region of the electromagnetic spectrum. It detects the absorption of light by molecular bonds and can be used with live insects. In this study, we investigate the accuracy of NIR spectroscopy in determining triglyceride level and species of wild‐caught *Drosophila*. We employ the chemometric approach to produce a multivariate calibration model. The multivariate calibration model is the mathematical relationship between the changes in NIR spectra and the property of interest as determined by the reference analytical method. Once the calibration model was developed, we used an independent set to validate the accuracy of the calibration model. The optimized calibration model for triglyceride quantification yielded coefficients of determination of 0.73 for the calibration test set and 0.70 for the independent test set. Simultaneously, we used NIR spectroscopy to discriminate two species of *Drosophila*. Flies from independent sets were correctly classified into *Drosophila melanogaster* and *Drosophila simulans* with accuracy higher than 80%. These results suggest that NIRS has the potential to be used as a high‐throughput screening method to assess a live individual insect's triglyceride level and taxonomic status.

## 1 INTRODUCTION

Near‐infrared spectroscopy (NIRS) detects the “chemical fingerprint” of a sample by measuring the amount of near‐infrared energy absorbed by biological materials at specific wavelengths (Álvarez‐Sánchez et al., 2013). The absorption is influenced by the internal and external chemical composition of the organism and is mainly generated from the stretching and bending of O–H, N–H, and C–H functional groups (Williams & Norris, 2001). Previously, we successfully employed NIRS to determine the species, gender, age, and the presence of *Wolbachia* infections in laboratory *Drosophila* (Aw, Dowell, & Ballard, 2012). There are at least five advantages of using NIRS in entomological research. First, it allows simultaneous analysis of multiple components from a single spectrum. Second, the operating cost for NIRS is low as no reagents or sample‐specific preparations are needed. Third, NIRS is a high‐throughput method to analyze NIR spectra in which more than 1,000 samples can be scanned per day. Fourth, NIRS technology is noninvasive and does not require a highly skilled technician for the operation of the instrument or the analysis of the acquired data after optimization and development of a calibration. Fifth, living organisms can be sampled.

In this study, we investigate the accuracy of NIRS in determining metabolites levels by comparing the results to those obtained from a commercial assay kit. NIRS has been applied in noninvasive measurement of a variety of metabolites including blood glucose of patients with type I diabetes (Robinson et al., 1992), less invasive quantitative measurement of lactate in humans (Lafrance, Lands, & Burns, 2003), and measurement of glucose, triglycerides, and high‐density lipoprotein of rat plasma (Neves, 2012). In insects, triglycerides constitute the main lipid form, representing ∼90% of the total fat body (Arrese, Patel, & Soulages, 2006). The content of triglycerides is influenced by several factors, including development stage, nutritional state, sex, and flight activity. Currently, triglyceride levels in insects are measured by commercial assay kits, gas chromatography–mass spectroscopy, and liquid chromatography–mass spectroscopy (Tennessen, Barry, Cox, & Thummel, 2014). Undesirably, all these technologies are costly, invasive, time‐consuming, and destructive.

Additionally, we test the accuracy of NIRS to correctly identify wild‐caught *Drosophila melanogaster *and *Drosophila simulans *by comparing it to an allele‐specific PCR test. There is a need for developing accurate, effective, low‐cost and efficient approaches that can be used in the field (Falk, Wallace, & Ndoen, 2011; Nansen & Elliott, 2016). Increasingly, NIRS is being used by the entomological community and it has been shown to accurately identify a range of species including *Anopheles *mosquitoes (Mayagaya et al., 2015), *Zootermopsis *termites (Aldrich, Maghirang, Dowell, & Kambhampati, 2007), *Calliphoridae *blowflies (Voss, Magni, Dadour, & Nansen, 2017), and *Tetramorium* ants (Kinzner et al., 2015). Morphologically, male *D. melanogaster* can be differentiated from male *D. simulans* by the shape of the genital arch genitalia, but females are difficult to identify and these taxa are considered sibling species. A biochemical approach is to use PCR with direct sequencing or allele‐specific PCR. However, the processing time and reagent costs often limit their application. As a consequence, field studies of wild‐caught *Drosophila* may be based upon a subsample of collected individuals, which may not capture the true heterogeneity of the sample.

In this study, we employ the chemometric strategy. Chemometric analysis is defined as the development and application of mathematical and statistical methods to extract useful chemical information from sample measurement (Gould, 1977). In the chemometric analysis, the best multivariate calibration model is obtained through step‐by‐step optimization compared to a known reference. The calibration model is the mathematical relationship between the changes in NIR spectra and the property of interest as determined by the reference analytical method (e.g., regression of measured absorption against reference analyte concentration data). In general, the sample size for a typical calibration model ranges between 40 and 90 samples, with smaller sample sizes potentially overfitting the data (Lafrance et al., 2003; Schulz, Drews, Quilitzsch, & Krüger, 1998). This calibration model is then tested with an independent data set, which includes samples not included in the developing of calibration model, to estimate its predictive ability (Mayagaya et al., 2009; Williams & Norris, 2001).

The aim of this study was to develop and validate a high‐throughput NIRS methodology for assessing the triglyceride levels and taxonomic status of wild‐caught *Drosophila*. We were able to determine triglyceride levels with a coefficient of determination of 0.70 and species with greater than 80% accuracy. Combined these results suggest that NIRS has the potential to be used as a high‐throughput screening method to assess a live individual insect's triglyceride levels and species status.

## 2 MATERIALS AND METHODS

### 2.1 NIRS scan of wild‐caught *Drosophila*

**Figure 1:**
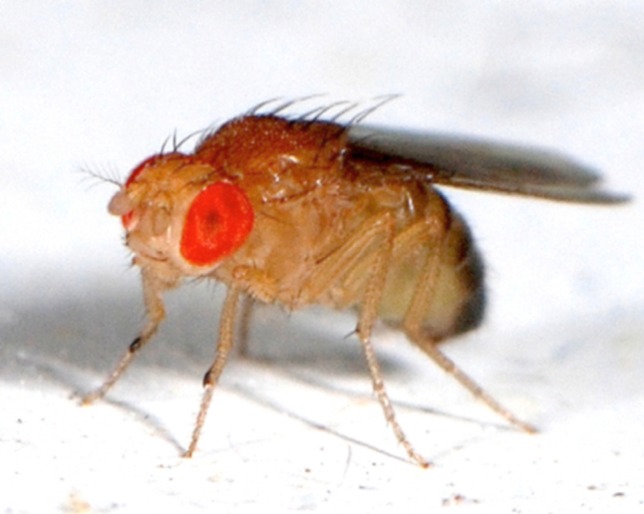
*Drosophila *is a genus of flies belonging to the family Drosophilidae. A male with visible sex combs on the forelegs is shown

Wild‐caught *Drosophila* flies (Figure 1) were collected in Rosebery, NSW, Australia, on six different days (27 February 2017–8 March 2017). Flies were placed in an empty vial and scanned using NIRS within 3 hr of collection. To ensure flies did not move during the NIRS scan, they were anesthetized with humidified CO_2_ for 30 min immediately before the scan was performed. The long CO_2 _sedation did not kill the flies but could cause metabolic changes (Colinet & Renault, 2012).

**Figure 2:**
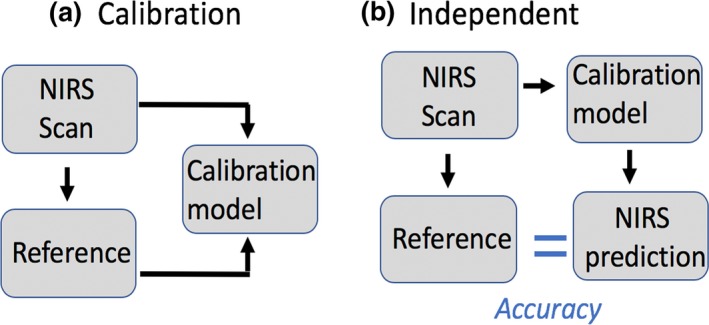
Strategy for metabolite quantification and species identification. (a) Calibration set included 159 wild‐caught flies. Flies were scanned using a NIR spectrometer and then frozen. For the reference, triglyceride content of 65 was determined by an assay kit. For species identification, the taxonomic status of 94 was determined by allele‐specific PCR. Chemometric analyses were employed to calculate calibration models. (b) Independent set included 121 flies. Again, flies were scanned using a NIR spectrometer and then frozen. The triglyceride level and species status of each fly were then predicted by the calibration model. For the reference, triglyceride levels were determined from 47 flies using the triglyceride assay kit, and species status of 74 adults was determined by the allele‐specific PCR. The accuracy of the calibration model was estimated by comparing the NIRS predicted value from the calibration model with the reference value (blue equal symbol)

The scanning system setup follows Mayagaya et al. (2009). About 25 flies were placed on a spectralon plate (ASD Inc., Boulder, Colorado, USA), and each fly was sexed and then individually scanned. The flies were placed 2 mm below a 3 mm diameter bifurcated fiber‐optic reflectance probe that contained 33 illumination fibers and 4 collection fibers. The probe was focused on the dorsal axis of the flies, and the spectra were collected with a portable LabSpec 5,000 spectrometer (350–2,500 nm; ASD Inc., Boulder, Colorado, USA) using RS^3^ Spectra Acquisition Software 6.0.10 (ASD Inc., Boulder, Colorado, USA). The raw channel data sampling rate of 1.4 nm in the visible and near‐infrared region (350–1,000 nm) and 2.2 nm in the short wavelength infrared region (1,001–2,500 nm) are interpolated to 1 nm intervals across the full spectrometer range from 350 nm to 2,500 nm. The nominal spectral resolution varies with the spectrometer region. The visible and near‐infrared region has a spectral resolution of 3 nm at 700 nm, and the short wavelength infrared region has a spectral resolution of 10 nm at 1,400 nm and 2,100 nm. An average of 50 spectra was collected from each sample and stored as an average spectrum. All spectra were converted into SPC format by the Asd to Spc convertor version 6 (ASD. Inc.). The spectra were then transformed into log 1/R and mean centered before analysis. After NIRS scanning, flies were frozen in liquid nitrogen and then transferred into a −80°C freezer (Figure 2).

### 2.2 REFERENCE

Triglyceride level and species status were independently determined. It was not possible to complete both assays on a single fly because both assays were performed through destructive wet chemistry analytical techniques (fluorometric kit and allele‐specific PCR). Due to this destructive sampling, we have limited sample sizes. A second limitation of the study is that it is assumed the reference data were obtained without error. This is unlikely completely true for the triglyceride assay because it is a continuous variable. It is more likely true for the allelic PCR because it was a discrete assay (it was either *D. melanogaster* or *D. simulans*) and independent validation corroborated 100% accuracy (Supporting Information Figure S2).

#### 2.2.1 Metabolite quantification

Reference triglyceride levels were determined using the Abcam fluorometric kit (AB65336) following the manufacturer's instructions. Briefly, triglycerides were hydrolyzed to free fatty acids and glycerol. The glycerol reacts with the triglyceride enzyme mix to form an intermediate product, which in turn reacted with the PicoProbe and developer to generate fluorescence that can be detected at Ex/Em = 537/587 nm. Experimental samples were prepared by grinding each adult fly in 100 µl of 5% NP‐40/ddH_2_O. Samples were slowly heated to 85°C for 5 min and then cooled down to room temperature. The heating and cooling process was repeated twice, and samples were centrifuged for 2 min at 4,000 *g* to remove insoluble materials. Triglyceride level was determined in a 384‐well microplate, with each well containing 25 µl of samples and 25 µl of working buffer (the working buffer consists of 23.8 µl of triglyceride assay buffer, 0.2 µl of triglyceride probe, and 1 µl of triglyceride enzyme mix). All measures were performed at 23°C. Triglyceride level was expressed as nmol/well (Supporting Information Figure S1).

#### 2.2.2 Species identification

Reference *Drosophila* species data were determined with allele‐specific PCR (Supporting Information Figure S2). The primers used for allele‐specific PCR were species‐specific and were designed by downloading and aligning 42 *cytochrome c oxidase I* (*cox I*) sequences from GenBank (15 *D. melanogaster* and 27 *D. simulans*). *Cox I *is a mitochondrial DNA‐encoded gene which is recognized as a DNA barcode, capable of accurate species identification in a broad range of animals (Hebert, Cywinska, Ball, & deWaard, 2003). The primers were validated with DNA samples from known species of laboratory flies, and the PCR products were sequenced to confirm the specificity of the primers. *D*. *simulans* was identified by amplifying a 784 bp region of *cox I* gene using primers 1856F (5′‐ TATCTGCTGGAATTGCCCAC‐3′) and 2642R (5′‐ GCTATAATAGCAAATACAGCTCC‐3′), while *D*. *melanogaster* was identified by amplifying a 600 bp region of *cox I* gene using primers 2041F (5′‐ GCTTTATTATTATTATTATCACTT‐3′) and 2642R (5′‐ GCTATAATAGCAAATACAGCTCC‐3′). Briefly, two sets of primer pairs were run in separate reactions and the allele was identified based on the band size on a gel. DNA was extracted from flies using a Gentra Puregene^®^ Cell kit (Gentra Sytem Inc., Minneapolis, MN, USA). Each 10 µl reaction contained 2 µl of Crimson™ buffer (NEB, New England Biolabs), 2.56 µl of 25 mM MgCl_2_, 0.4 µl of 10 mM forward and reverse primer, 0.08 µl of 25 mM dNTP, 0.05 µl of Taq polymerase, and 2.51 µl of H_2_O and 10 ng of DNA. The PCR cycling program involved four separate phases. Phase 1 was the initial denaturation which was 94°C for 2 min. Second, the 5 cycle touchdown phase (denaturation: 94°C for 10 s, annealing: 64°C for 15 s with the temperature gradually reducing 1°C per cycle until it reached 59°C, and extension: 1 min at 72°C). Third, the 20 cycle phase (denaturation 94°C for 10 s, annealing 59°C for 15 s and 1 min at 72°C). Fourth, a final 72°C extension step for 6 min.

### 2.3 Calibration models

The NIR scan and the reference data (triglyceride and allele‐specific PCR) were individually paired to develop four calibration models. One calibration model was developed for the triglyceride quantification, and three models were developed for species identification. The calibration models were constructed with partial least square (PLS) regression leave‐one‐out cross‐validation method using GRAMS IQ version 9.1 (Thermo Fisher Scientific, Salem, NH; Williams & Norris, 2001). PLS regression analysis was calculated to determine the quantitative relation between raw near‐infrared spectra and chemical composition of the sample. Cross‐validation is carried out by dividing the population of samples into equal “blocks” and eliminating samples one block at a time. Consequently, all samples were used in the development of the calibration equation. This technique is appropriate for small sample sizes. A regression coefficient plot was used to analyze PLS models for each composition and to determine noises region in the model. This plot shows noise increases outside 500–2,200 nm, and these regions were excluded.

All spectra were smoothed using the Savitzky–Golay first derivative method (Savitzky & Golay, 1964). Calculating derivatives of spectral data by the Savitzky–Golay numerical algorithm is a widely used pretreatment method that can effectively resolve overlapping signals, enhance signal properties, and suppress unwanted spectral features that arise from nonideal instrument and sample properties (Chen, Song, Tang, Feng, & Lin, 2013; Zimmermann & Kohler, 2013). Considering the vast range of possible signal bandwidths encountered within a typical spectrum, it is not possible for us to employ a general smoothing function with set parameters for spectral preprocessing using the Savitzky–Golay procedure. Therefore, we optimize each model independently. The point smoothing function with maximum accuracy in the independent test set was chosen. The outlier samples were identified by Mahalanobis distance (Mahalanobis, 1936). There were less than 5% outliers in all models.

#### 2.3.1 Metabolite quantification

A calibration model was generated using 65 wild‐collected flies (Figure 1a). In the model, the spectra were assigned with the reference value obtained from the fluorometric assay. All spectra were processed using the Savitzky–Golay first derivative with 35 point smoothing function. We did not develop sex‐specific models because sample sizes were less than 40, and this may have resulted in the calibration model overfitting the data (Lafrance et al., 2003; Schulz et al., 1998).

#### 2.3.2 Species identification

An initial calibration model (Model 1) was developed using the mixture of 94 male and female flies (Figure 1a). To determine whether the accuracy of species identification could be improved when sexes are considered separately, we then divided the samples into males and females and then developed sex‐specific models (Guan et al., 2013). Model 2 was a calibration model for the 50 males. Model 3 was a calibration model for the 44 females. Necessarily, the sex‐specific models reduced the sample size, which concomitantly increases the likelihood of overfitting the data. As such, caution should be exercised in interpreting the results from Models 2 and 3.

In all the calibration models, the spectra of *D. melanogaster *flies were assigned a value of 1, and *D. simulans *were assigned a value of 2. The value of 1.5 was considered as the cutoff point for species identification. Flies with predicted value less than 1.5 were classified as *D. melanogaster*, whereas those with a predicted value equal to or greater than 1.5 were classified as *D. simulans*. All spectra were processed using the Savitzky–Golay first derivative with 5 point smoothing function.

### 2.4 Independent set

To minimize the potential problem of calibration model's overfitting the data, we included an independent data set. If a model developed fit to the training set also fits the test set well, minimal overfitting has taken place (Subramanian & Simon, 2013). The independent sets were analyzed using GRAMS IQ Predict version 9.1 (Thermo Galactic, Salem, NH).

#### 2.4.1 Metabolite quantification

The independent set was generated using 47 wild‐collected flies. The predicted value for triglyceride level was then determined using the metabolite calibration model and compared with that determined by the fluorometric assay.

#### 2.4.2 Species identification

Three independent sets were used to estimate the accuracy of each calibration model. Independent set 1 was developed using both male and female flies (74 flies). Independent set 2 included males (44 flies) and set 3 females (30 flies). Spectra with species identified using allele‐specific PCR were then compared with the three calibration models.

### 2.5 Accuracy

Accuracy represents the combination of the sum of the trueness (systematic error) and precision (random error; Baratloo, Hosseini, Negida, & Ashal, 2015). Here, the best multivariate calibration model was chosen based on the highest accuracy of prediction of the independent set.

#### 2.5.1 Accuracy of metabolite quantification

The optimized calibration model for triglyceride quantification was chosen based on coefficients of determination. Triglyceride levels determined from the fluorescent kit were continuous, and the accuracy of the triglyceride level quantification was determined by measuring the root‐mean‐square error of calibration, coefficient of determination (*R* ^2^), root‐mean‐square error of prediction (RMSEP), and the ratio of the standard error of performance to the standard deviation of the reference data (RPD). Root‐mean‐square error of calibration (RMSEC) and *R* ^2^ were used to measure goodness of fit between the reference data and the calibration model. The RMSEP and the RPD, computed from the independent set, were used to measure the differences between the predicted value and the reference value. The closer the predicted scan result is to the actual or known result the lower the RMSEP value and the higher the RPD. A good model should have lower RMSEC, lower RMSEP, higher RPD, and higher *R* ^2^. To enable comparison with the species identification results, we focus upon *R* ^2^ as a measure of accuracy.

#### 2.5.2 Accuracy of species identification

Species identification using allele‐specific PCR was discrete (1 for *D. melanogaster* and 2 for *D. simulans*), and accuracy could be determined as a percentage. The accuracy was calculated by comparing the allelic‐specific PCR result with the scan result. The closer the predicted result is to the allelic‐specific PCR result the greater the accuracy.

## 3 RESULT AND DISCUSSION

### 3.1 Metabolite quantification

**Figure 3:**
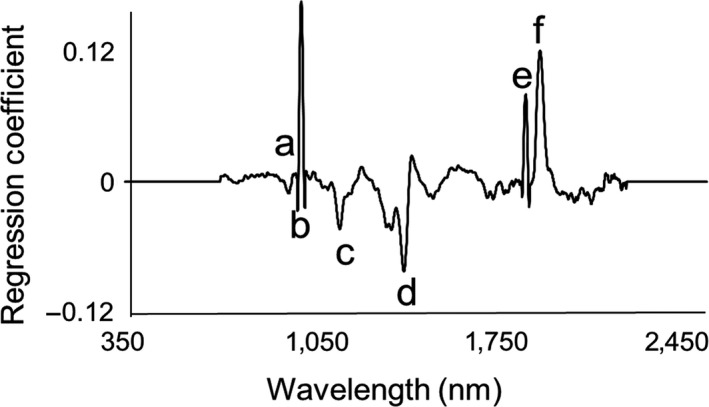
Regression coefficient plot for triglyceride quantification was generated with eight partial least square regression factors. (a) 920 nm, (b) 1,040 nm, (c) 1,140 nm, (d) 1,370 nm, (e) 1,820 nm, and (f) 1,900 nm

The regression coefficient plot for triglyceride quantification showed peaks in the regions around 920 nm, 1,040 nm, 1,140 nm, 1,370 nm, 1820 nm, and 1900 nm (Figure 3), which were consistent with the absorptions of functional groups associated with glycerol and fatty acids. These functional groups include methyl group (–CH_3_), methylene group (–CH_2_), alkene group (C=C), and ester group (COOC). The peaks at 920 nm, 1,040 nm, 1,140 nm, and 1,370 nm are characteristic of the 2nd overtone and the combination of C–H stretching. Notably, similar absorbance coefficient peaks around 920 nm and 1,040 nm were also observed in tissue samples with high‐fat content (ElMasry & Nakauchi, 2016; Wilson, Nadeau, Jaworski, Tromberg, & Durkin, 2015). The peak at 1,820 nm shows the 1st overtone of C–H stretching, whereas peak on 1,900 nm corresponds to the absorption of COOC functional groups (Williams & Norris, 2001).

**Figure 4:**
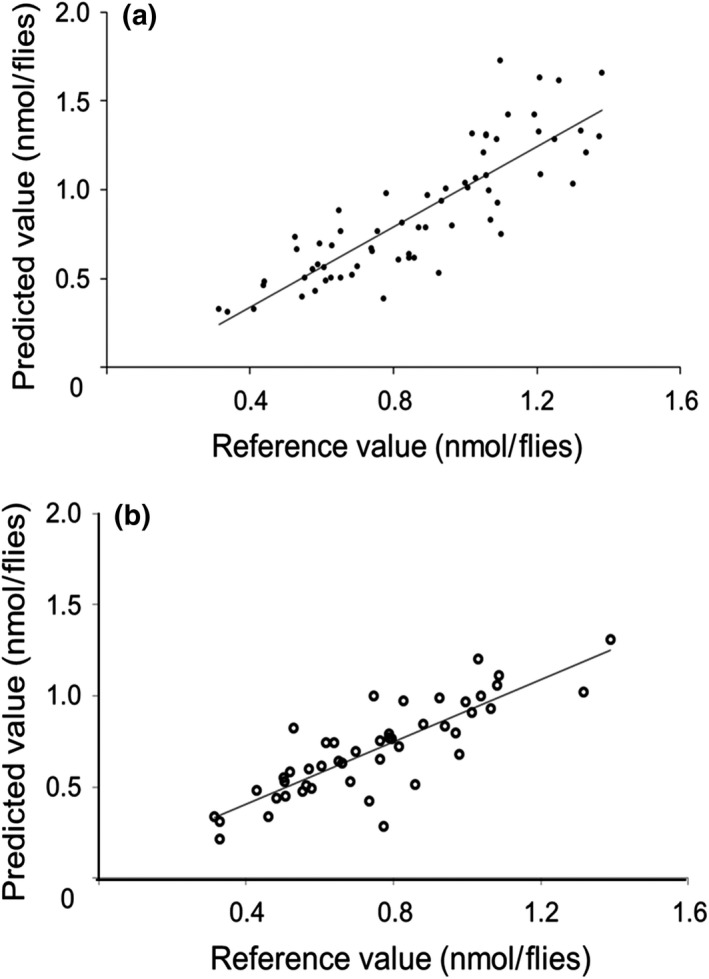
Relationship between the reference fluorometric kit and the NIRS predicted triglyceride values in the calibration set and independent test set. (a) The calibration model has a coefficient of determination of 0.73 for the calibration set. (b) The calibration model has a coefficient of determination of 0.70 for the independent test set

Reference triglyceride concentrations of the wild‐caught flies were determined using a commercial fluorometric kit and ranged between 0.312 and 1.526 nmol/fly (Supporting Information Figure S1). The optimized calibration model for triglyceride quantification yields a RMSEC of 0.19, RMSEP value of 0.26, and RPD of 1.92. An RPD of 1.92 indicated poor NIR reflectance predictions (Williams & Norris, 2001). Considering male and female flies can differ drastically in their metabolite level (Rong et al., 2014), future studies should increase the sample size and then developed sex‐specific models to increase the efficiency of NIR predictions. The calibration model has a *R* ^2^ of 0.73 and 0.70 for the calibration set and independent test set, respectively (Figure 4). In contrast to our results, Neves et al. (2012) developed a NIRS calibration model with a correlation coefficient of 0.96 for triglyceride quantification in animal plasma.

### 3.2 Species identification

**Figure 5:**
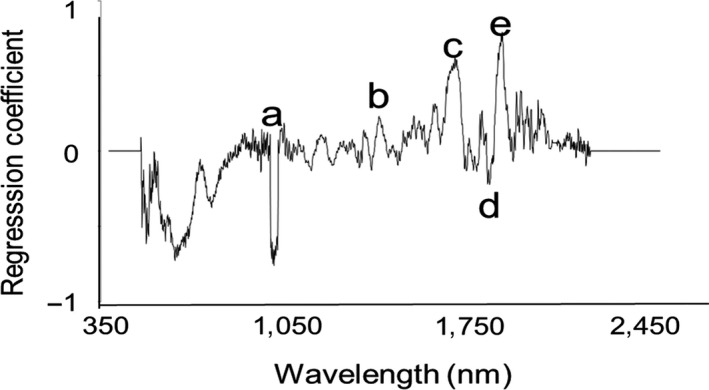
Regression coefficient plot for classifying *Drosophila melanogaster *and *Drosophila simulans* was generated with six partial least square regression factors. (a) 1,040 nm, (b) 1,450 nm, (c) 1,720 nm, (d) 1,820 nm, and (e) 1,900 nm

The regression coefficient plot show peaks in the regions around 1,040, 1,450, 1,720, 1,820, and 1,900 nm (Figure 5). Notably, the peak around 1,450 nm was observed in our previous study on species identification of laboratory *Drosophila *(Aw et al., 2012). The peak at 1,450 nm is characteristics of the 1st overtone and the combination of C–H stretching and has been shown to increase with the rise in moisture content of the sample (Yang et al., 2013). Peaks at 1,040, 1,820, and 1,900 nm were observed in the regression coefficient plot for triglyceride quantification (Figure 3) but not observed in the species identification of laboratory *Drosophila *(Aw et al., 2012). This implies that lipid may play an important role in species discrimination of the wild‐caught flies but are less important in the species identification of laboratory flies raised in a standard diet. This finding is consistent with Fischnaller, Dowell, Lusser, Schlick‐Steiner, and Steiner (2012) who showed that validation sets obtained from wild‐caught flies cannot be apply to laboratory‐reared flies, and vice versa.

**Figure 6:**
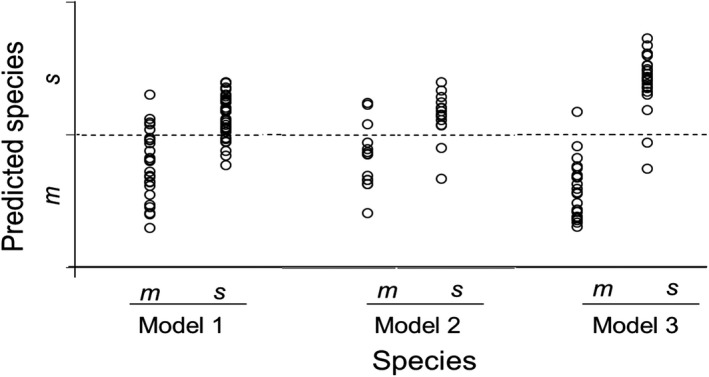
NIRS species identification of *Drosophila melanogaster *(*m*) and *Drosophila simulans *(*s*) in the independent test set using three calibration models. Dotted line indicated cutoff point for delineating species. Calibration Model 1 includes all flies. Calibration Model 2 includes male flies. Calibration Model 3 includes females

In this study, we categorized the wild‐caught flies as either *D. melanogaster *or *D. simulans* using allele‐specific PCR (Supporting Information Figure S2). Model 1 developed using the mixture of both sexes correctly classified flies as *D. melanogaster *and *D. simulans *with 80% (*n* = 74) accuracy. Dividing the samples into males and females improved the accuracy of species identification. In calibration Model 2, male flies were correctly classified as *D. melanogaster *and *D. simulans *with 93.2% accuracy. Model 3 correctly identified female flies as *D. melanogaster* and *D. simulans* with 83.35% accuracy (Figure 6). The optimized calibration model for species identification of wild‐caught *Drosophila* (Figure 6) and of laboratory‐reared *Drosophila *(Aw et al., 2012) had greater than 80% accuracy of prediction. Similarly, the NIRS calibration model for two field‐collected mosquito species also had an accuracy of approximately 80% (Mayagaya et al., 2009). In comparison, the accuracy of identifying four *Tetramorium* ant species was lower (13.3%–66.7%; Kinzner et al., 2015).

## 4 CONCLUSION

The current methods for metabolite quantification and sibling species identification can be difficult and laborious. To overcome these difficulties, we tested the potential use of NIRS for triglyceride quantification and species identification. The major advantages of NIRS technique for entomologists include the cost saving after initial purchase of the instrument, nondestructive sampling, and the potential for high‐throughput analysis. Our study demonstrated NIRS can quantify triglyceride with an *R* ^2^ of 0.70 and identify wild‐caught *Drosophila* with an accuracy of higher than 80%. The major limitation is that the methodology is not 100% accurate. In cases where very high accuracy is required, NIRS may be able to provide an initial screening of the data as the specimens are not damaged.

Ongoing goals are to increase the accuracy and usage of NIRS. Here, we show that the accuracy of species identification improved when calibration models were independently developed for males and females. Necessarily, this reduced our overall sample sizes. Future studies should include a sufficient number of samples so that calibration models can be independently developed for males and for females. Future studies may also include perturbation assays and simulations so optimal sample sizes can be determined and biases associated with over fitting the data can be determined. Additional challenges include linking additional metabolites with NIRS spectral patterns and simultaneously identifying more than two species. Kinzner et al. (2015) demonstrate that four species of ant (*Tetramorium*) could be classified by NIRS using one versus all strategy with an accuracy of 13%–67% (Rifkin & Klautau, 2004). We conclude that NIRS is a promising method for monitoring of insect's metabolite level and taxonomic status, and further optimization may well improve the accuracy of the technique.

## AUTHOR CONTRIBUTIONS

Conceived and designed the experiment: W.C.A and J.W.O.B. Performed the experiment: W.C.A. Analyzed data: W.C.A. Wrote manuscript: W.C.A. and J.W.O.B. All authors contributed critically to the drafts and gave final approval for publication.

## Data Availability

The raw data and spectra have been submitted to Dryad and can be viewed at https://doi.org/10.5061/dryad.324ch00.
