# Supplementary figures and images for: Near‐infrared spectroscopy for metabolite quantification and species identification
Source: Ecol Evol. 2019 Jan 13;9(3):1336–43. doi: 10.1002/ece3.4847 (PMC6374719; doi:10.1002/ece3.4847)

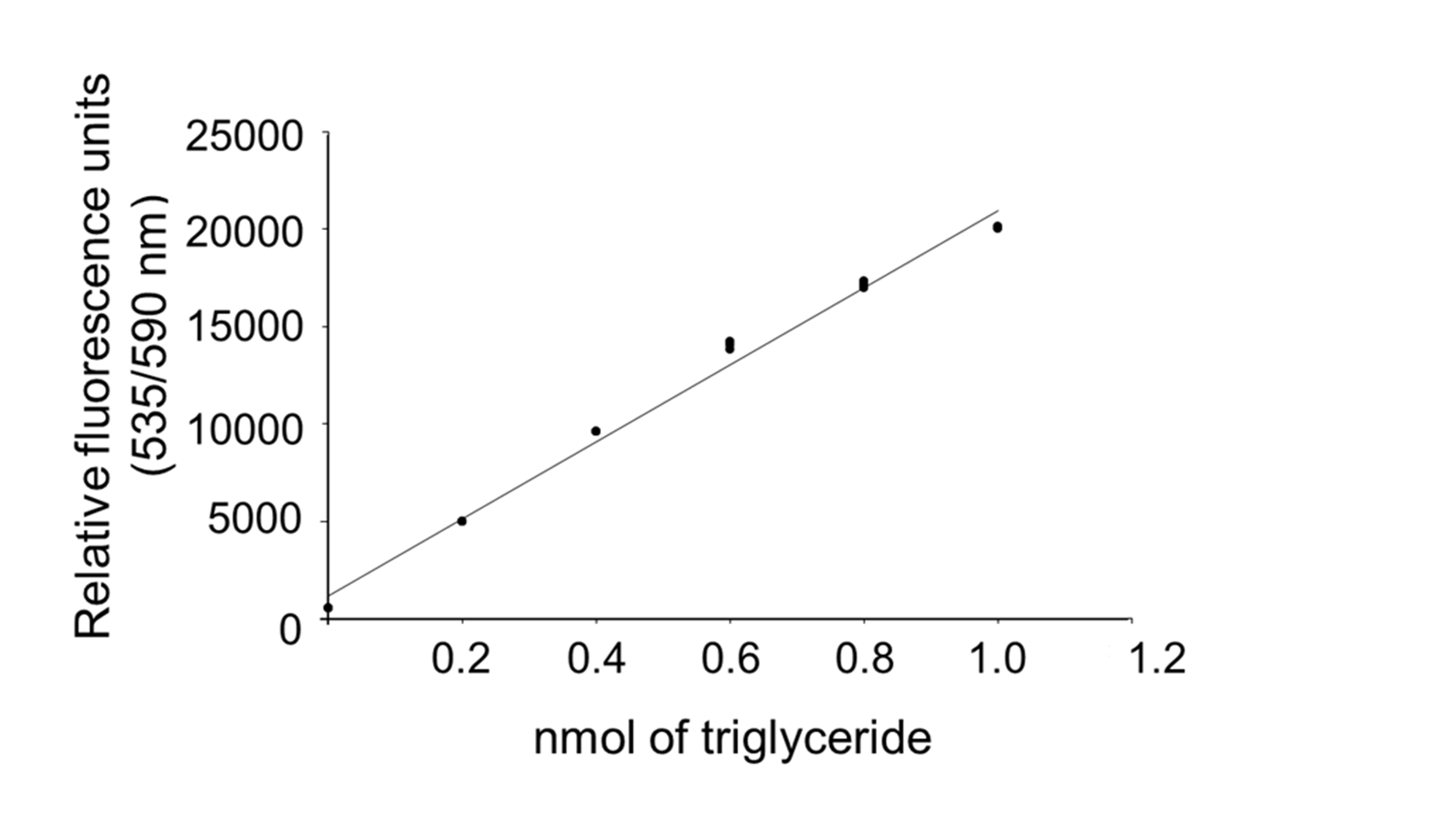

Supplement: Supplementary file 1 [file ECE3-9-1336-s001.tif]

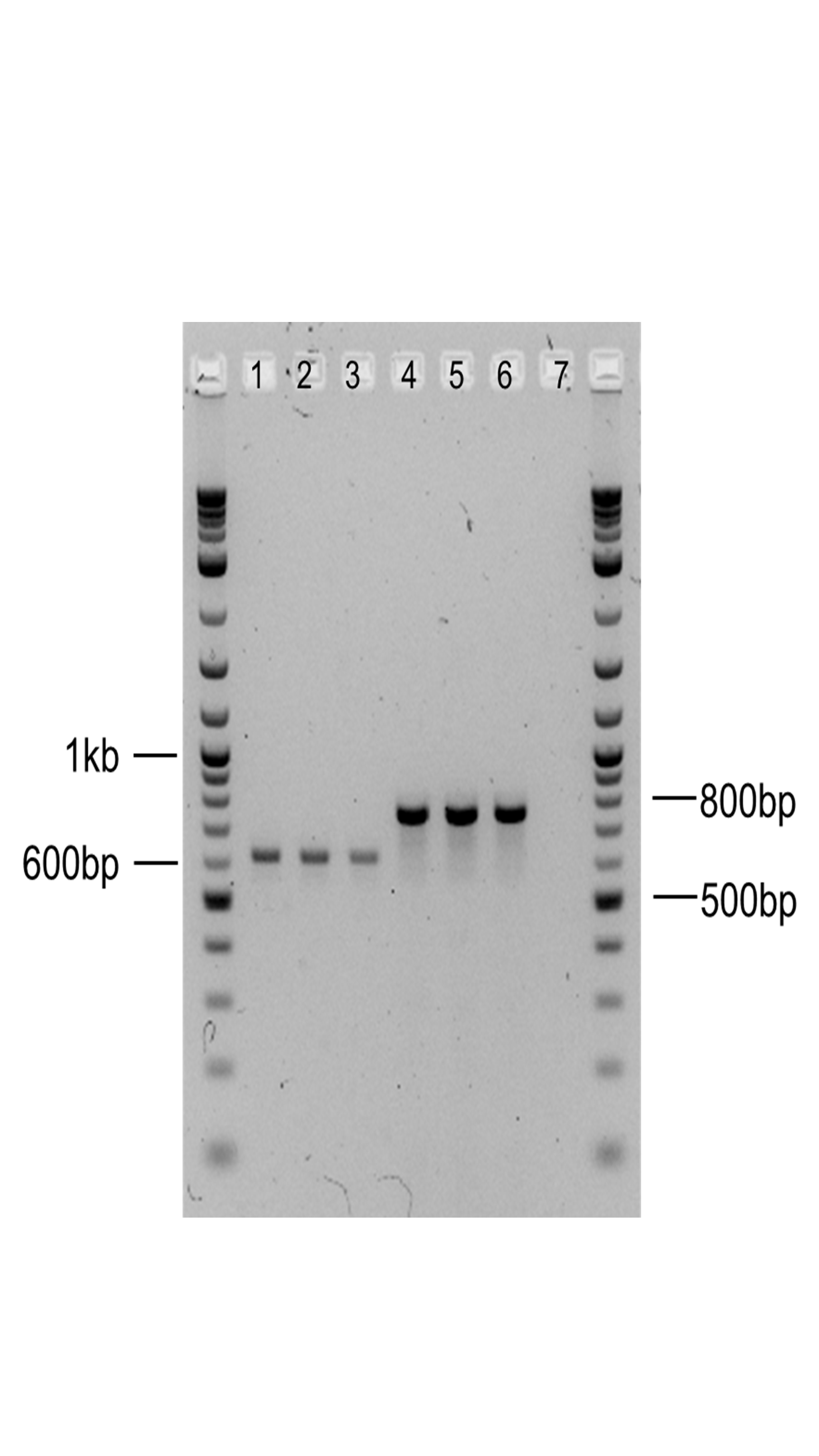

Supplement: Supplementary file 2 [file ECE3-9-1336-s002.tif]
